# Anomalous Cases of Tabes Dorsalis

**Published:** 1902-08-16

**Authors:** 


### ANOMALOUS CASES OF TABES DORSALIS.

A case of tabes dorsalis in which the symptoms are fairly typical in each of the several directions in which they usually display themselves, does not generally present any particular difficulties in diagnosis, since it has been from the observation of such cases that the clinical history of the disease as given in the text-books has been built up. Nevertheless, true as may be the generally accepted picture of the disease, it is to be noted that, even within that group of cases in which the various symptoms are all well marked, a great variation is to be observed in regard to their relative intensity in different cases; sometimes the eye symptoms, sometimes the ataxy, and sometimes the crises may predominate, even in cases where they are all present. But other cases occur in which certain parts seem to be affected almost to the exclusion of the rest; indeed, one is almost led to the belief that there is in some cases a sort of inverse ratio between the local intensity and the widespread diffusion of those destructive changes to which the symptoms are due.

In a very interesting clinical lecture delivered at the National Hospital on July 8th, Dr. James Taylor [1] dealt with certain anomalous cases of tabes, the most important of which were, firstly, those in which symptoms referable to the eye were the chief manifestation of the disease, and, secondly, those characterised by the obtrusiveness of gastric crises. Cases in the first group, in their most common form, have either paralysis of some ocular muscle or they have optic atrophy. A slight though quite definite degree of double ptosis is by no means uncommon in cases of tabes without any involvement of any other muscle. Or there may be paralysis of one or both sixth nerves—most frequently one. Curiously enough these ocular paralyses occurring in cases of tabes are nearly always of a transient character, and clear up, usually, under the influence of iodide and mercury. Possibly such cases are not always definitely tabetic, but as a rule other symptoms can be found which indicate their relations. There may, for example, be pupils which are inactive to light, or lightning pains, sometimes in the distribution of the fifth nerve, or girdle sensation, or loss of knee-jerk, and true tabetic symptoms, although at first in abeyance, may afterwards become fully developed.

Again, in some cases of grey atrophy there may, in the early stage, be no other symptom than a slight degree of optic atrophy with a corresponding defect of vision. There may not even be loss of knee-jerk, although a few months later one may find that the knee-jerk has disappeared, and that the patient is having occasional attacks of shooting pains of moderate intensity in the arms or legs, or in both. Still, again, ocular symptoms may take the form of Argyll-Robertson pupil, which may stand alone, being, except for a slight inequality in the size of the pupils, the only sign of definite importance. Such cases are nearly always of the tabetic type, and sooner or later develop some unequivocal sign of degeneration corresponding to the disease.

A point of some clinical interest is, that in most tabetics who have optic atropy, ataxy is not present. This is especially true of cases in which failure of vision from optic atrophy is an early symptom. So evident is this, that it has often been stated that the onset of optic atrophy in a tabetic patient is followed by an arrest of the progress of the disease. This dictum, however, probably does not properly express the fact, for marked ataxy may develop in cases of optic atrophy, and, without doubt, cases may occur in which the onset of optic atrophy has no effect in modifying the progression of the symptoms. Yet, taking the mass of the cases, it is no doubt true that in tabetic patients with optic atrophy, especially those in whom this is severe and the affection of vision is profound, the other usual symptoms, and particularly the ataxy, are either absent or only present in a very mild form.

We come now to a sometimes very puzzling set of cases, namely, those in which gastric or other crises are accompanied by none of the other ordinary signs of tabes, such as ataxy, lightning pains, or pupillary changes. Such cases are very apt to be regarded as merely "bilious," and to go on for a long time without their true nature being discovered. In a case related the patient was a healthy, strong-looking man of 35, who complained of occasional "bilious attacks." These attacks of vomiting were characterised by their extremely sudden onset, by the fact that they lasted but two or three days, severe vomiting being accompanied by excessive prostration, and that they passed away almost as suddenly as they came. The pupils reacted perfectly, there was no ataxy, no interference with the action of the sphincters, and the patient was able to do very exacting professional work. The only indication of tabes besides the crises was that no knee-jerk could be elicited on either side. Such cases are probably by no means uncommon, and Dr. Taylor insists that in every case in which, in an apparently healthy man, there is a history of recurrent attacks of severe vomiting, one should not be content with a diagnosis of "bilious attack," but should search for symptoms of serious nervous disease. As for the after history of these cases, as in those of optic atrophy, there may be no development of ataxy, and Dr. Taylor, speaking from his own experience, is inclined to say that the predominance of gastric crises, even if there are other undoubted symptoms of tabetic lesion present, may encourage one to hope that the patient is not likely to become ataxic, and that, as a matter of fact, the gastric crises may entirely cease and the disease afterwards remain, at all events during many years, without any perceptible advance. Thus we have here another illustration of the fact that an abnormally partial distribution of tabetic symptoms is apt to be associated with a mode of progress of the disease which differs from the ordinary clinical type. In regard to the treatment of these cases, Dr. Taylor says that there is nothing which controls a gastric crisis when it has fairly established itself except the hypodermic use of morphine, but, lest the morphine habit should be set up, it is essential that the administration should not be left in the hands of the patient himself. He has not been encouraged by what he has seen of the action of antipyrin and that class of drugs, but says that it is possible that the energetic use of one or other of them, combined perhaps with large doses of bismuth and a small quantity of morphine by the mouth, might have a beneficial effect.

[1] Brit. Med. Jour., July 19.

